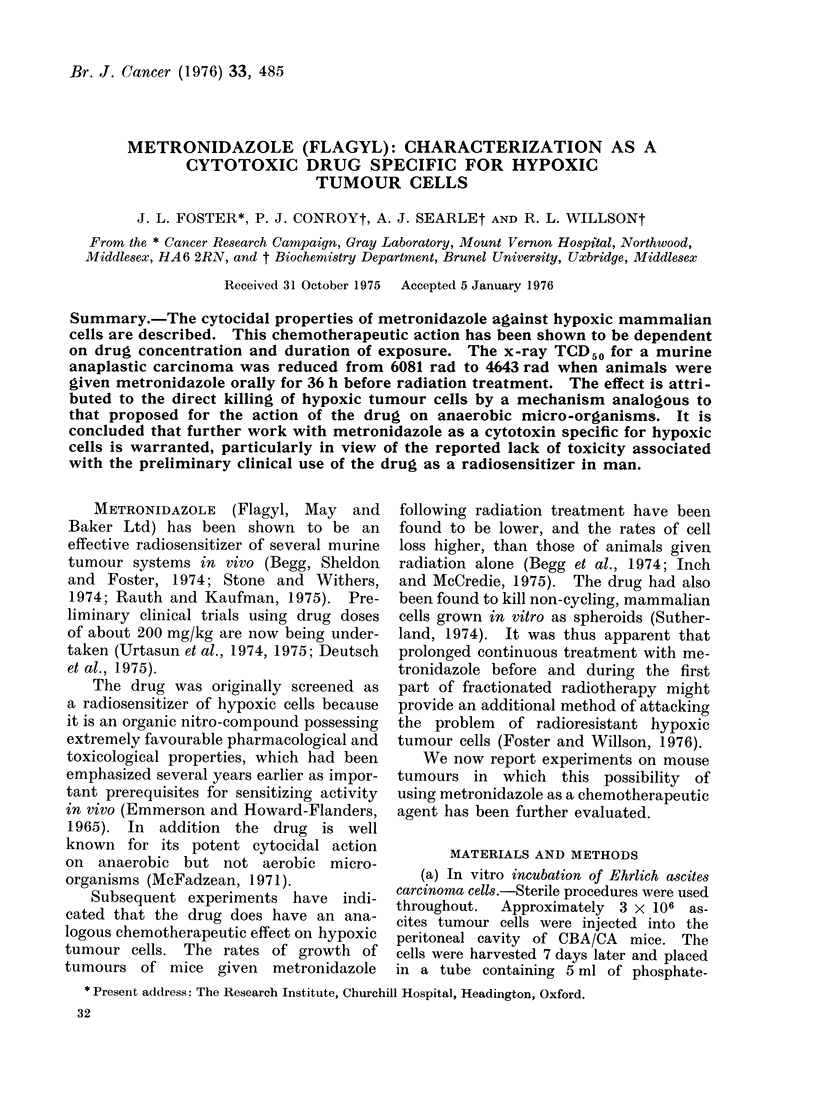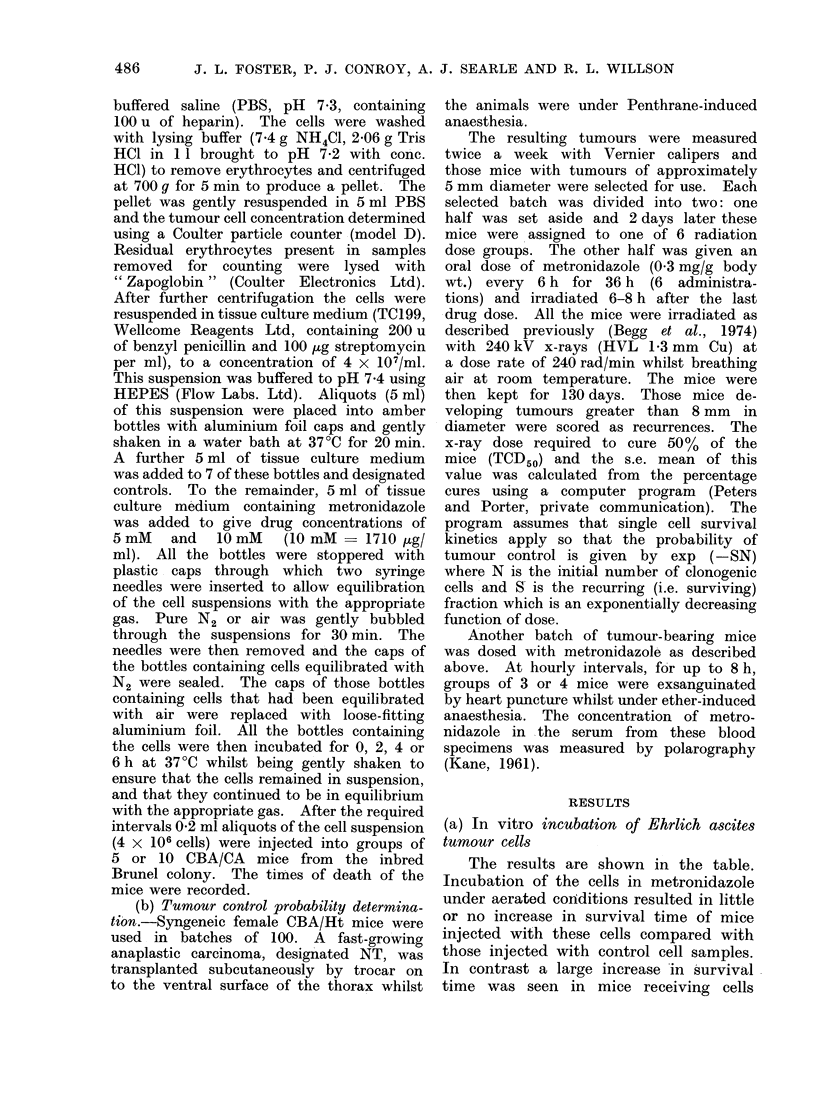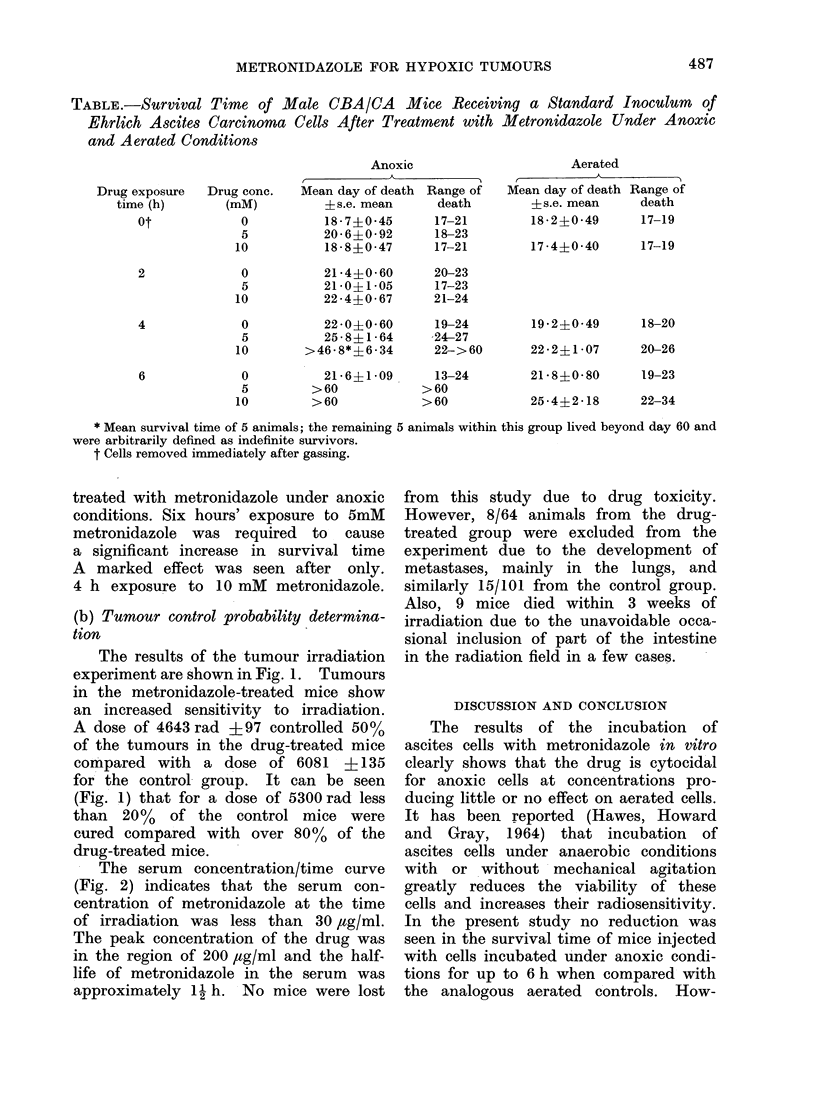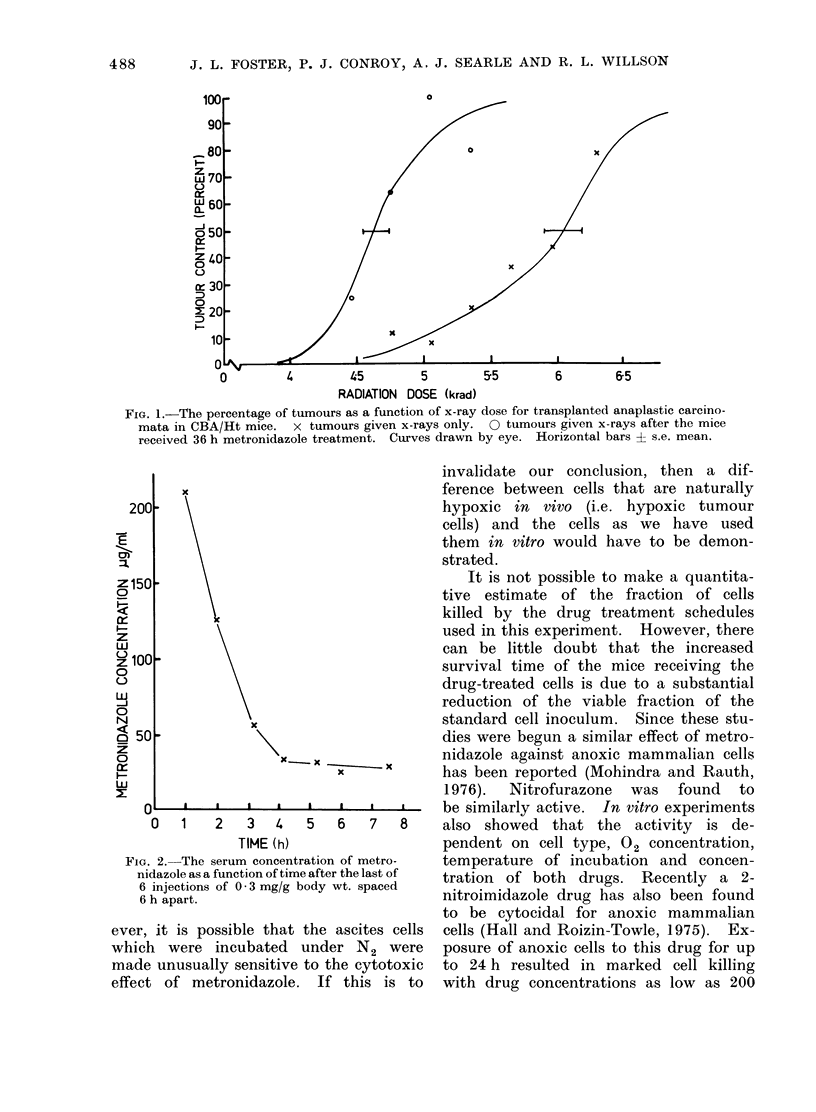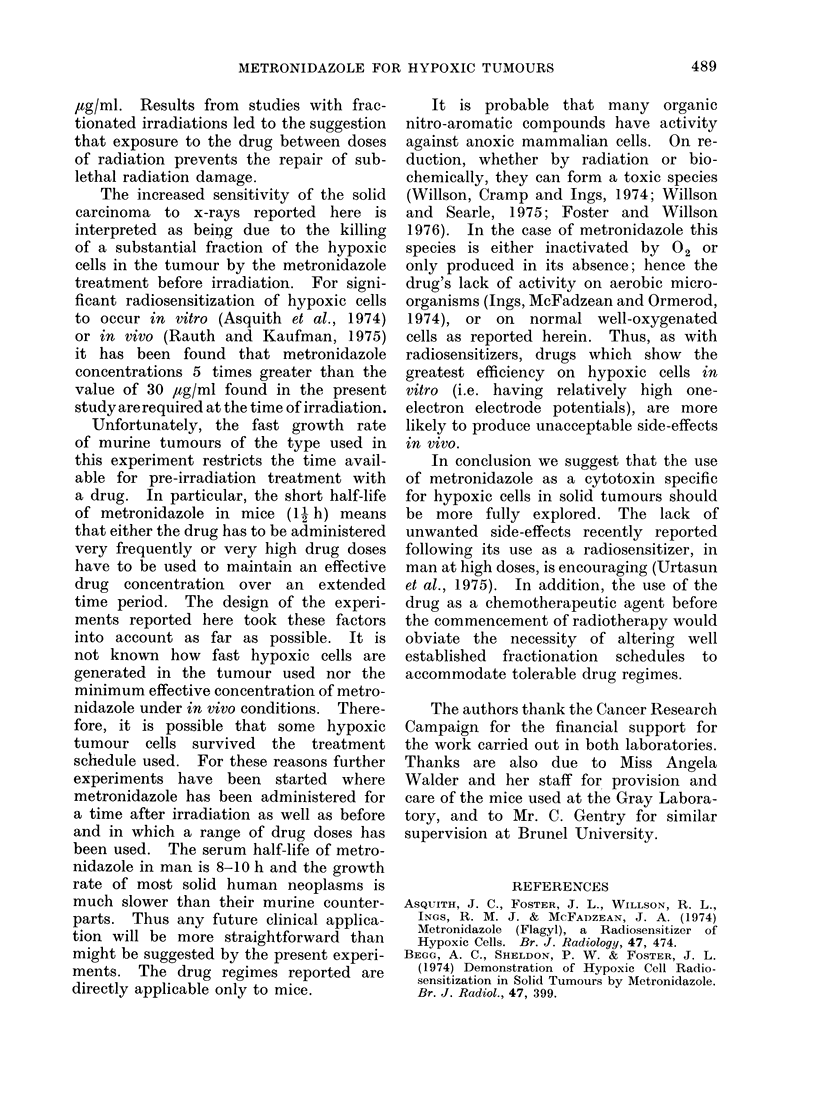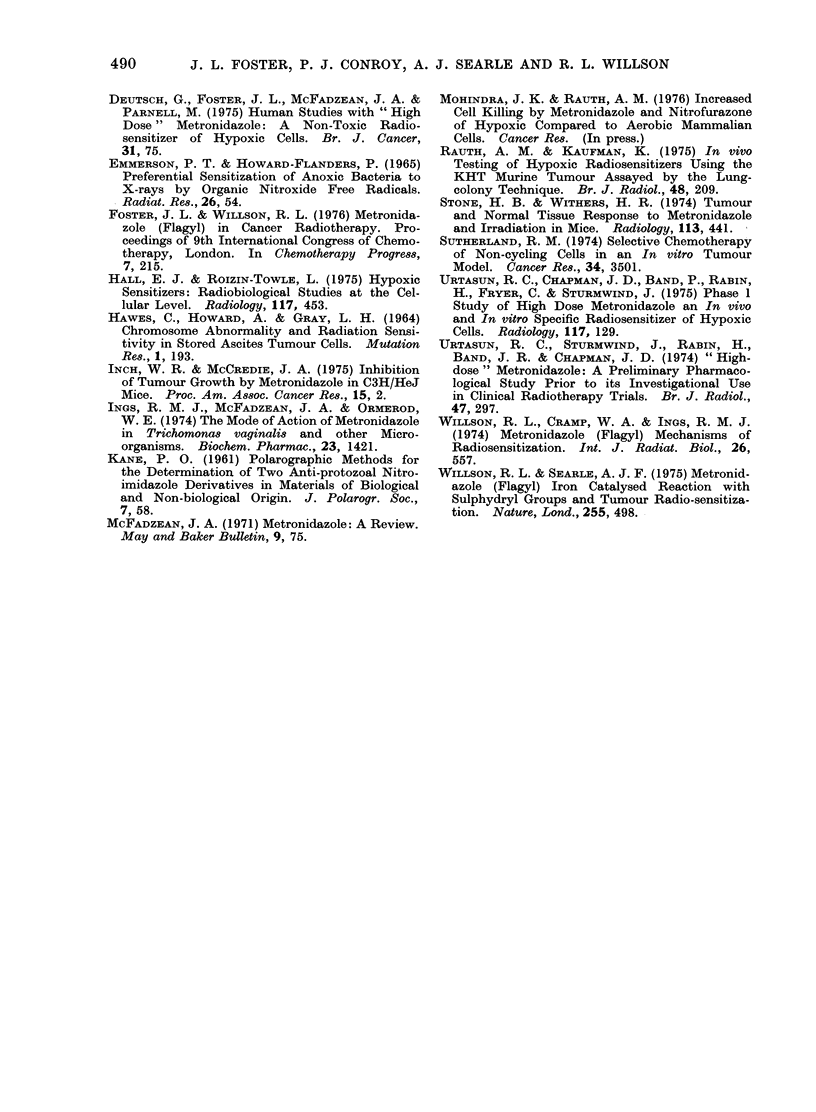# Metronidazole (Flagyl): characterization as a cytotoxic drug specific for hypoxic tumour cells.

**DOI:** 10.1038/bjc.1976.78

**Published:** 1976-05

**Authors:** J. L. Foster, P. J. Conroy, A. J. Searle, R. L. Willson

## Abstract

The cytocidal properties of metronidazole against hypoxic mammalian cells are described. This chemotherapeutic action has been shown to be dependent on drug concentration and duration of exposure. The x-ray TCD50 for a murine anaplastic carcinoma was reduced from 6081 rad to 4643 rad when animals were given metronidazole orally for 36 h before radiation treatment. The effect is attributed to the direct killing of hypoxic tumour cells by a mechanism analogous to that proposed for the action of the drug on anaerobic micro-organisms. It is concluded that further work with metronidazole as a cytotoxin specific for hypoxic cells is warranted, particularly in view of the reported lack of toxicity associated with the preliminary clinical use of the drug as a radiosensitizer in man.


*Br. J. Cancer* (1976) **33**, 485

### METRONIDAZOLE (FLAGYL): CHARACTERIZATION AS A CYTOTOXIC DRUG SPECIFIC FOR HYPOXIC TUMOUR CELLS

J. L. FOSTER*, P. J. CONROY†, A. J. SEARLE† AND R. L. WILLSON†

*From the * Cancer Research Campaign, Gray Laboratory, Mount Vernon Hospital, Northwood, Middlesex, HA6 2RN, and † Biochemistry Department, Brunel University, Uxbridge, Middlesex*

Received 31 October 1975 Accepted 5 January 1976

**Summary.—The cytocidal properties of metronidazole against hypoxic mammalian cells are described. This chemotherapeutic action has been shown to be dependent on drug concentration and duration of exposure. The x-ray $TCD_{50}$ for a murine anaplastic carcinoma was reduced from 6081 rad to 4643 rad when animals were given metronidazole orally for 36 h before radiation treatment. The effect is attributed to the direct killing of hypoxic tumour cells by a mechanism analogous to that proposed for the action of the drug on anaerobic micro-organisms. It is concluded that further work with metronidazole as a cytotoxin specific for hypoxic cells is warranted, particularly in view of the reported lack of toxicity associated with the preliminary clinical use of the drug as a radiosensitizer in man.**

METRONIDAZOLE (Flagyl, May and Baker Ltd) has been shown to be an effective radiosensitizer of several murine tumour systems *in vivo* (Begg, Sheldon and Foster, 1974; Stone and Withers, 1974; Rauth and Kaufman, 1975). Preliminary clinical trials using drug doses of about 200 mg/kg are now being undertaken (Urtasun *et al.*, 1974, 1975; Deutsch *et al.*, 1975).

The drug was originally screened as a radiosensitizer of hypoxic cells because it is an organic nitro-compound possessing extremely favourable pharmacological and toxicological properties, which had been emphasized several years earlier as important prerequisites for sensitizing activity *in vivo* (Emmerson and Howard-Flanders, 1965). In addition the drug is well known for its potent cytocidal action on anaerobic but not aerobic micro-organisms (McFadzean, 1971).

Subsequent experiments have indicated that the drug does have an analogous chemotherapeutic effect on hypoxic tumour cells. The rates of growth of tumours of mice given metronidazole following radiation treatment have been found to be lower, and the rates of cell loss higher, than those of animals given radiation alone (Begg *et al.*, 1974; Inch and McCredie, 1975). The drug had also been found to kill non-cycling, mammalian cells grown *in vitro* as spheroids (Sutherland, 1974). It was thus apparent that prolonged continuous treatment with metronidazole before and during the first part of fractionated radiotherapy might provide an additional method of attacking the problem of radioresistant hypoxic tumour cells (Foster and Willson, 1976).

We now report experiments on mouse tumours in which this possibility of using metronidazole as a chemotherapeutic agent has been further evaluated.

### MATERIALS AND METHODS

(a) In vitro *incubation of Ehrlich ascites carcinoma cells.*—Sterile procedures were used throughout. Approximately $3 \times 10^6$ ascites tumour cells were injected into the peritoneal cavity of CBA/CA mice. The cells were harvested 7 days later and placed in a tube containing 5 ml of phosphate-

* Present address: The Research Institute, Churchill Hospital, Headington, Oxford.

buffered saline (PBS, pH 7·3, containing 100 u of heparin). The cells were washed with lysing buffer (7·4 g $NH_4Cl$, 2·06 g Tris HCl in 1 l brought to pH 7·2 with conc. HCl) to remove erythrocytes and centrifuged at 700 *g* for 5 min to produce a pellet. The pellet was gently resuspended in 5 ml PBS and the tumour cell concentration determined using a Coulter particle counter (model D). Residual erythrocytes present in samples removed for counting were lysed with "Zapoglobin" (Coulter Electronics Ltd). After further centrifugation the cells were resuspended in tissue culture medium (TC199, Wellcome Reagents Ltd, containing 200 u of benzyl penicillin and 100 μg streptomycin per ml), to a concentration of $4 \times 10^7$/ml. This suspension was buffered to pH 7·4 using HEPES (Flow Labs. Ltd). Aliquots (5 ml) of this suspension were placed into amber bottles with aluminium foil caps and gently shaken in a water bath at 37°C for 20 min. A further 5 ml of tissue culture medium was added to 7 of these bottles and designated controls. To the remainder, 5 ml of tissue culture medium containing metronidazole was added to give drug concentrations of 5 mM and 10 mM (10 mM = 1710 μg/ml). All the bottles were stoppered with plastic caps through which two syringe needles were inserted to allow equilibration of the cell suspensions with the appropriate gas. Pure $N_2$ or air was gently bubbled through the suspensions for 30 min. The needles were then removed and the caps of the bottles containing cells equilibrated with $N_2$ were sealed. The caps of those bottles containing cells that had been equilibrated with air were replaced with loose-fitting aluminium foil. All the bottles containing the cells were then incubated for 0, 2, 4 or 6 h at 37°C whilst being gently shaken to ensure that the cells remained in suspension, and that they continued to be in equilibrium with the appropriate gas. After the required intervals 0·2 ml aliquots of the cell suspension ($4 \times 10^6$ cells) were injected into groups of 5 or 10 CBA/CA mice from the inbred Brunel colony. The times of death of the mice were recorded.

(b) *Tumour control probability determination.*—Syngeneic female CBA/Ht mice were used in batches of 100. A fast-growing anaplastic carcinoma, designated NT, was transplanted subcutaneously by trocar on to the ventral surface of the thorax whilst the animals were under Penthrane-induced anaesthesia.

The resulting tumours were measured twice a week with Vernier calipers and those mice with tumours of approximately 5 mm diameter were selected for use. Each selected batch was divided into two: one half was set aside and 2 days later these mice were assigned to one of 6 radiation dose groups. The other half was given an oral dose of metronidazole (0·3 mg/g body wt.) every 6 h for 36 h (6 administrations) and irradiated 6–8 h after the last drug dose. All the mice were irradiated as described previously (Begg *et al.*, 1974) with 240 kV x-rays (HVL 1·3 mm Cu) at a dose rate of 240 rad/min whilst breathing air at room temperature. The mice were then kept for 130 days. Those mice developing tumours greater than 8 mm in diameter were scored as recurrences. The x-ray dose required to cure 50% of the mice ($TCD_{50}$) and the s.e. mean of this value was calculated from the percentage cures using a computer program (Peters and Porter, private communication). The program assumes that single cell survival kinetics apply so that the probability of tumour control is given by exp (—SN) where N is the initial number of clonogenic cells and S is the recurring (i.e. surviving) fraction which is an exponentially decreasing function of dose.

Another batch of tumour-bearing mice was dosed with metronidazole as described above. At hourly intervals, for up to 8 h, groups of 3 or 4 mice were exsanguinated by heart puncture whilst under ether-induced anaesthesia. The concentration of metronidazole in the serum from these blood specimens was measured by polarography (Kane, 1961).

### RESULTS

### (a) In vitro *incubation of Ehrlich ascites tumour cells*

The results are shown in the table. Incubation of the cells in metronidazole under aerated conditions resulted in little or no increase in survival time of mice injected with these cells compared with those injected with control cell samples. In contrast a large increase in survival time was seen in mice receiving cells

TABLE.—*Survival Time of Male CBA/CA Mice Receiving a Standard Inoculum of Ehrlich Ascites Carcinoma Cells After Treatment with Metronidazole Under Anoxic and Aerated Conditions*

| | | Anoxic | | Aerated | |
|---|---|---|---|---|---|
| Drug exposure time (h) | Drug conc. (mM) | Mean day of death ±s.e. mean | Range of death | Mean day of death ±s.e. mean | Range of death |
| 0† | 0 | 18·7±0·45 | 17–21 | 18·2±0·49 | 17–19 |
| | 5 | 20·6±0·92 | 18–23 | | |
| | 10 | 18·8±0·47 | 17–21 | 17·4±0·40 | 17–19 |
| 2 | 0 | 21·4±0·60 | 20–23 | | |
| | 5 | 21·0±1·05 | 17–23 | | |
| | 10 | 22·4±0·67 | 21–24 | | |
| 4 | 0 | 22·0±0·60 | 19–24 | 19·2±0·49 | 18–20 |
| | 5 | 25·8±1·64 | 24–27 | | |
| | 10 | >46·8*±6·34 | 22–>60 | 22·2±1·07 | 20–26 |
| 6 | 0 | 21·6±1·09 | 13–24 | 21·8±0·80 | 19–23 |
| | 5 | >60 | >60 | | |
| | 10 | >60 | >60 | 25·4±2·18 | 22–34 |

* Mean survival time of 5 animals; the remaining 5 animals within this group lived beyond day 60 and were arbitrarily defined as indefinite survivors.
† Cells removed immediately after gassing.

treated with metronidazole under anoxic conditions. Six hours' exposure to 5mM metronidazole was required to cause a significant increase in survival time A marked effect was seen after only. 4 h exposure to 10 mM metronidazole.

(b) *Tumour control probability determination*

The results of the tumour irradiation experiment are shown in Fig. 1. Tumours in the metronidazole-treated mice show an increased sensitivity to irradiation. A dose of 4643 rad ±97 controlled 50% of the tumours in the drug-treated mice compared with a dose of 6081 ±135 for the control group. It can be seen (Fig. 1) that for a dose of 5300 rad less than 20% of the control mice were cured compared with over 80% of the drug-treated mice.

The serum concentration/time curve (Fig. 2) indicates that the serum concentration of metronidazole at the time of irradiation was less than 30 μg/ml. The peak concentration of the drug was in the region of 200 μg/ml and the half-life of metronidazole in the serum was approximately 1½ h. No mice were lost from this study due to drug toxicity. However, 8/64 animals from the drug-treated group were excluded from the experiment due to the development of metastases, mainly in the lungs, and similarly 15/101 from the control group. Also, 9 mice died within 3 weeks of irradiation due to the unavoidable occasional inclusion of part of the intestine in the radiation field in a few cases.

### DISCUSSION AND CONCLUSION

The results of the incubation of ascites cells with metronidazole *in vitro* clearly shows that the drug is cytocidal for anoxic cells at concentrations producing little or no effect on aerated cells. It has been reported (Hawes, Howard and Gray, 1964) that incubation of ascites cells under anaerobic conditions with or without mechanical agitation greatly reduces the viability of these cells and increases their radiosensitivity. In the present study no reduction was seen in the survival time of mice injected with cells incubated under anoxic conditions for up to 6 h when compared with the analogous aerated controls. How-

FIG. 1.—The percentage of tumours as a function of x-ray dose for transplanted anaplastic carcinomata in CBA/Ht mice. × tumours given x-rays only. ○ tumours given x-rays after the mice received 36 h metronidazole treatment. Curves drawn by eye. Horizontal bars ± s.e. mean.

FIG. 2.—The serum concentration of metronidazole as a function of time after the last of 6 injections of 0·3 mg/g body wt. spaced 6 h apart.

ever, it is possible that the ascites cells which were incubated under $N_2$ were made unusually sensitive to the cytotoxic effect of metronidazole. If this is to invalidate our conclusion, then a difference between cells that are naturally hypoxic *in vivo* (i.e. hypoxic tumour cells) and the cells as we have used them *in vitro* would have to be demonstrated.

It is not possible to make a quantitative estimate of the fraction of cells killed by the drug treatment schedules used in this experiment. However, there can be little doubt that the increased survival time of the mice receiving the drug-treated cells is due to a substantial reduction of the viable fraction of the standard cell inoculum. Since these studies were begun a similar effect of metronidazole against anoxic mammalian cells has been reported (Mohindra and Rauth, 1976). Nitrofurazone was found to be similarly active. *In vitro* experiments also showed that the activity is dependent on cell type, $O_2$ concentration, temperature of incubation and concentration of both drugs. Recently a 2-nitroimidazole drug has also been found to be cytocidal for anoxic mammalian cells (Hall and Roizin-Towle, 1975). Exposure of anoxic cells to this drug for up to 24 h resulted in marked cell killing with drug concentrations as low as 200

μg/ml. Results from studies with fractionated irradiations led to the suggestion that exposure to the drug between doses of radiation prevents the repair of sublethal radiation damage.

The increased sensitivity of the solid carcinoma to x-rays reported here is interpreted as being due to the killing of a substantial fraction of the hypoxic cells in the tumour by the metronidazole treatment before irradiation. For significant radiosensitization of hypoxic cells to occur *in vitro* (Asquith *et al.*, 1974) or *in vivo* (Rauth and Kaufman, 1975) it has been found that metronidazole concentrations 5 times greater than the value of 30 μg/ml found in the present study are required at the time of irradiation.

Unfortunately, the fast growth rate of murine tumours of the type used in this experiment restricts the time available for pre-irradiation treatment with a drug. In particular, the short half-life of metronidazole in mice ($1\frac{1}{2}$ h) means that either the drug has to be administered very frequently or very high drug doses have to be used to maintain an effective drug concentration over an extended time period. The design of the experiments reported here took these factors into account as far as possible. It is not known how fast hypoxic cells are generated in the tumour used nor the minimum effective concentration of metronidazole under *in vivo* conditions. Therefore, it is possible that some hypoxic tumour cells survived the treatment schedule used. For these reasons further experiments have been started where metronidazole has been administered for a time after irradiation as well as before and in which a range of drug doses has been used. The serum half-life of metronidazole in man is 8–10 h and the growth rate of most solid human neoplasms is much slower than their murine counterparts. Thus any future clinical application will be more straightforward than might be suggested by the present experiments. The drug regimes reported are directly applicable only to mice.

It is probable that many organic nitro-aromatic compounds have activity against anoxic mammalian cells. On reduction, whether by radiation or biochemically, they can form a toxic species (Willson, Cramp and Ings, 1974; Willson and Searle, 1975; Foster and Willson 1976). In the case of metronidazole this species is either inactivated by $O_2$ or only produced in its absence; hence the drug's lack of activity on aerobic micro-organisms (Ings, McFadzean and Ormerod, 1974), or on normal well-oxygenated cells as reported herein. Thus, as with radiosensitizers, drugs which show the greatest efficiency on hypoxic cells *in vitro* (i.e. having relatively high one-electron electrode potentials), are more likely to produce unacceptable side-effects *in vivo*.

In conclusion we suggest that the use of metronidazole as a cytotoxin specific for hypoxic cells in solid tumours should be more fully explored. The lack of unwanted side-effects recently reported following its use as a radiosensitizer, in man at high doses, is encouraging (Urtasun *et al.*, 1975). In addition, the use of the drug as a chemotherapeutic agent before the commencement of radiotherapy would obviate the necessity of altering well established fractionation schedules to accommodate tolerable drug regimes.

The authors thank the Cancer Research Campaign for the financial support for the work carried out in both laboratories. Thanks are also due to Miss Angela Walder and her staff for provision and care of the mice used at the Gray Laboratory, and to Mr. C. Gentry for similar supervision at Brunel University.

### REFERENCES

Asquith, J. C., Foster, J. L., Willson, R. L., Ings, R. M. J. & McFadzean, J. A. (1974) Metronidazole (Flagyl), a Radiosensitizer of Hypoxic Cells. *Br. J. Radiology*, **47**, 474.

Begg, A. C., Sheldon, P. W. & Foster, J. L. (1974) Demonstration of Hypoxic Cell Radiosensitization in Solid Tumours by Metronidazole. *Br. J. Radiol.*, **47**, 399.

DEUTSCH, G., FOSTER, J. L., MCFADZEAN, J. A. & PARNELL, M. (1975) Human Studies with "High Dose" Metronidazole: A Non-Toxic Radiosensitizer of Hypoxic Cells. *Br. J. Cancer*, **31**, 75.

EMMERSON, P. T. & HOWARD-FLANDERS, P. (1965) Preferential Sensitization of Anoxic Bacteria to X-rays by Organic Nitroxide Free Radicals. *Radiat. Res.*, **26**, 54.

FOSTER, J. L. & WILLSON, R. L. (1976) Metronidazole (Flagyl) in Cancer Radiotherapy. Proceedings of 9th International Congress of Chemotherapy, London. In *Chemotherapy Progress*, 7, 215.

HALL, E. J. & ROIZIN-TOWLE, L. (1975) Hypoxic Sensitizers: Radiobiological Studies at the Cellular Level. *Radiology*, **117**, 453.

HAWES, C., HOWARD, A. & GRAY, L. H. (1964) Chromosome Abnormality and Radiation Sensitivity in Stored Ascites Tumour Cells. *Mutation Res.*, **1**, 193.

INCH, W. R. & MCCREDIE, J. A. (1975) Inhibition of Tumour Growth by Metronidazole in C3H/HeJ Mice. *Proc. Am. Assoc. Cancer Res.*, **15**, 2.

INGS, R. M. J., MCFADZEAN, J. A. & ORMEROD, W. E. (1974) The Mode of Action of Metronidazole in *Trichomonas vaginalis* and other Microorganisms. *Biochem. Pharmac.*, **23**, 1421.

KANE, P. O. (1961) Polarographic Methods for the Determination of Two Anti-protozoal Nitroimidazole Derivatives in Materials of Biological and Non-biological Origin. *J. Polarogr. Soc.*, **7**, 58.

MCFADZEAN, J. A. (1971) Metronidazole: A Review. *May and Baker Bulletin*, **9**, 75.

MOHINDRA, J. K. & RAUTH, A. M. (1976) Increased Cell Killing by Metronidazole and Nitrofurazone of Hypoxic Compared to Aerobic Mammalian Cells. *Cancer Res.* (In press.)

RAUTH, A. M. & KAUFMAN, K. (1975) *In vivo* Testing of Hypoxic Radiosensitizers Using the KHT Murine Tumour Assayed by the Lung-colony Technique. *Br. J. Radiol.*, **48**, 209.

STONE, H. B. & WITHERS, H. R. (1974) Tumour and Normal Tissue Response to Metronidazole and Irradiation in Mice. *Radiology*, **113**, 441.

SUTHERLAND, R. M. (1974) Selective Chemotherapy of Non-cycling Cells in an *In vitro* Tumour Model. *Cancer Res.*, **34**, 3501.

URTASUN, R. C., CHAPMAN, J. D., BAND, P., RABIN, H., FRYER, C. & STURMWIND, J. (1975) Phase 1 Study of High Dose Metronidazole an *In vivo* and *In vitro* Specific Radiosensitizer of Hypoxic Cells. *Radiology*, **117**, 129.

URTASUN, R. C., STURMWIND, J., RABIN, H., BAND, J. R. & CHAPMAN, J. D. (1974) "High-dose" Metronidazole: A Preliminary Pharmacological Study Prior to its Investigational Use in Clinical Radiotherapy Trials. *Br. J. Radiol.*, **47**, 297.

WILLSON, R. L., CRAMP, W. A. & INGS, R. M. J. (1974) Metronidazole (Flagyl) Mechanisms of Radiosensitization. *Int. J. Radiat. Biol.*, **26**, 557.

WILLSON, R. L. & SEARLE, A. J. F. (1975) Metronidazole (Flagyl) Iron Catalysed Reaction with Sulphydryl Groups and Tumour Radio-sensitization. *Nature, Lond.*, **255**, 498.